# Design and Evaluation of Potentiometric Principles for Bladder Volume Monitoring: A Preliminary Study

**DOI:** 10.3390/s150612802

**Published:** 2015-06-01

**Authors:** Shih-Ching Chen, Tsung-Hsun Hsieh, Wen-Jia Fan, Chien-Hung Lai, Chun-Lung Chen, Wei-Feng Wei, Chih-Wei Peng

**Affiliations:** 1 Department of Physical Medicine and Rehabilitation, School of Medicine, College of Medicine, Taipei Medical University, No. 250, Wuxing Street, Taipei 11031, Taiwan; E-Mails: csc@tmu.edu.tw (S.-C.C.); selectin1106@yahoo.com.tw (W.-J.F.); chlai@tmu.edu.tw (C.-H.L.); longdi1008@gmail.com (C.-L.C.); 2 Department of Physical Medicine and Rehabilitation, Taipei Medical University Hospital, No. 252, Wuxing Street, Taipei 11031, Taiwan; 3 Graduate Institute of Neural Regenerative Medicine, Taipei Medical University, No. 250, Wuxing Street, Taipei 11031, Taiwan; E-Mail: hsiehth@tmu.edu.tw; 4 Department of Physical Therapy and Graduate Institute of Rehabilitation Science, College of Medicine and Healthy Aging Research Center, Chang Gung University, No. 259, Wenhua 1st Rd, Taoyuan 33302, Taiwan; 5 Graduate Institute of Biomedical Electronics and Bioinformatics, National Taiwan University, No.1, Roosevelt Road, Sec.4, Taipei 10617, Taiwan; E-Mail: webberway@gmail.com

**Keywords:** potentiometer, real time, pig bladder, implantable, sensor

## Abstract

Recent advances in microelectronics and wireless transmission technology have led to the development of various implantable sensors for real-time monitoring of bladder conditions. Although various sensing approaches for monitoring bladder conditions were reported, most such sensors have remained at the laboratory stage due to the existence of vital drawbacks. In the present study, we explored a new concept for monitoring the bladder capacity on the basis of potentiometric principles. A prototype of a potentiometer module was designed and fabricated and integrated with a commercial wireless transmission module and power unit. A series of *in vitro* pig bladder experiments was conducted to determine the best design parameters for implementing the prototype potentiometric device and to prove its feasibility. We successfully implemented the potentiometric module in a pig bladder model *in vitro*, and the error of the accuracy of bladder volume detection was <±3%. Although the proposed potentiometric device was built using a commercial wireless module, the design principles and animal experience gathered from this research can serve as a basis for developing new implantable bladder sensors in the future.

## 1. Introduction

The primary functions of the lower urinary tract (LUT) are storage and periodic elimination of urine. These functions require reciprocal coordination of the urinary bladder and urethral outlet, including the bladder neck, urethra and external urethral sphincter (EUS). Neural pathways controlling bladder functions in humans are complex [1,2]. Bladder voiding dysfunction (urinary retention), developed as a result of aging, diabetes, spinal cord injury or complications after pelvic surgery, are usually accompanied by a permanent loss of bladder sensation [3,4]. This potentially induces an overdistension of the bladder, which may lead to serious complications, such as frequent urinary tract infections (UTIs), incontinence and high bladder pressure, causing reflux of urine to the ureters and kidneys. Urinary retention is often refractory to pharmacological, behavioral and surgical approaches [5], and many patients must resort to intermittent self-catheterization, which itself is associated with frequent UTIs and a reduced quality of life.

Neuromodulation is an alternative treatment for voiding dysfunction. Various neuromodulation implants, mainly using electrical stimulation to regulate reflex in bladder control, were proposed to effectively control storage and evacuation of urine [6,7]. To the best of our knowledge, none of the neuromodulation implants for bladder emptying currently used in clinical practice have a closed-loop control system. Therefore, neuromodulation for urine evacuation is conduct every 4–6 h following a fixed time schedule. However, if the emptying interval is not chosen properly, it will increase the risk of complications due to an overfull bladder, such as bladder overdistension injury, reflux, hydronephrosis or autonomic dysreflexia.

Laboratory catheterization-based urodynamics is the current standard for clinical urological examinations. This approach can accurately detect physiological conditions of the LUT, but it is difficult to use for long-term monitoring of a patient’s urological condition in an ambulatory environment or at home. Therefore, there is a growing interest in the development of various non-invasive and implantable sensors to monitor conditions of bladder volume or pressure, which can be utilized as feedback information to closed-loop control of neuromodulation implants or alert a patient to conduct intermittent self-catheterization at appropriate times. Different sensing techniques for real-time monitoring of bladder volumes have been proposed by researchers, including magnetism [8], impedance [9,10], ultrasonic [11,12], electroneurography (ENG) [13,14] and other measurements [15,16,17]. There are challenging factors due to the anatomic and physiologic characteristics of the LUT or other factors; thus, all of these proposed sensing schemes still remain at the laboratory stage [18,19,20].

The aim of this study was to explore a new approach for bladder volume assessment based on the principle of the potentiometer. In this proof-of-concept study, a prototype of a flexible potentiometer module was integrated with wireless transmission and power modules. A series of *in vitro* experiments was conducted to determine the best design parameters for implementing the prototype device and to prove the feasibility of the concept of the potentiometric sensor.

## 2. Experimental Section

### 2.1. Basic Structure of the Prototype Potentiometric Platform

**Figure 1:**
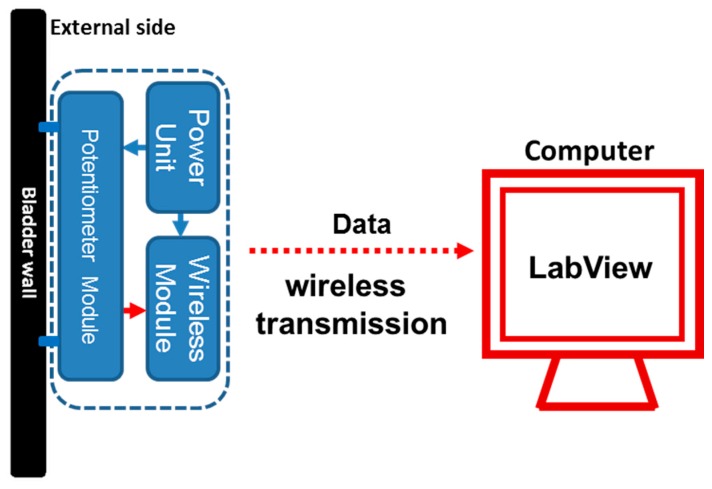
Schematic representation of the prototype potentiometric device for bladder volume monitoring. The power unit supplied a stable, constant 3-mA current to the flexible potentiometer and Bluetooth wireless transmission modules. The two ends of the flexible potentiometer were fixed to the outer bladder wall, where the resistance value of the potentiometer changed on the basis of changes in the bladder wall length. Then, the Bluetooth wireless transmission module, an Arduino Nano 3.0, sent the digital data to an external receiver of a computer via wireless transmission. The received data were further processed using the LabVIEW program, and the bladder capacity information was displayed on the computer screen in real time.

The potentiometric platform was composed of three parts: a flexible potentiometric module, a wireless transmission module and a power unit. Figure 1 depicts the overall structure of the sensing platform.

In this study, bladder capacity measurements were designed on the basis of the principle of a linear potentiometer attached to the bladder’s outer wall. The rationale for using a linear potentiometer was that the two terminals of the potentiometer are fixed on an object, and the potentiometer produces a resistance output that varies according to changes in the length of the object between the two terminals of the potentiometer. Commercially-available potentiometers are often used to detect length changes or position displacements of a linear object. However, those potentiometers are not suitable for bladder capacity measurements, because the bladder is a spherical structure, not a flat plane, and all commercial potentiometers are made of inflexible materials, which do not allow flexible bending to fit geometric changes in the bladder wall. Thus, in this study, a flexible potentiometer was made of fiberglass material of a printed circuit board (PCB). The advantages of the fiberglass material were that it had flexible properties, while also maintaining good hardness.

**Figure 2:**
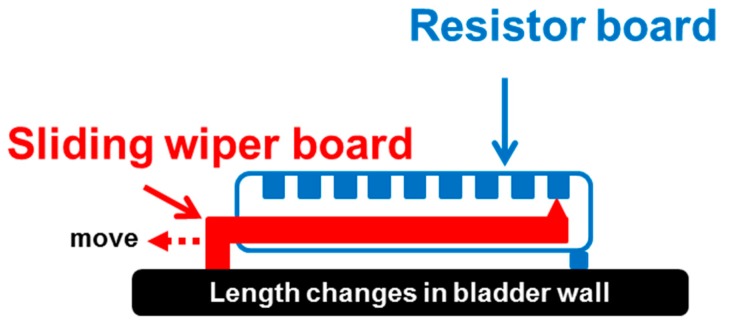
Conceptual drawing of the potentiometric module for detecting length changes in the bladder wall. The custom-made flexible potentiometer consisted of two fiberglass printed circuit boards (PCBs), including one board for potentiometric resistance that was soldered with resistors and the other board as a sliding wiper that contacted one of the resistors. The bladder capacity was predicted by the resistance value.

The custom-made flexible potentiometer consisted of two fiberglass PCBs, including one board used as for potentiometric resistance and the other board as a sliding wiper, as shown in Figure 2. The resistance board was soldered with surface-mounted device (SMD) resistors (Vishay Intertechnology Inc., Malvern, PA, USA). A small raised point was welded onto the sliding board, where the “bump” of the sliding board would contact each resistor of the resistance board to measure the value of the resistance (Figure 2). Therefore, the bladder capacity could be measured based on the resistance value of the potentiometric device. To validate the feasibility of the preliminary design of the potentiometer module, a power unit and wireless transmission module were integrated with the potentiometer module and tested on an *in vitro* pig bladder model.

### 2.2. Bladder Geometric Model

On the basis of a change in the length in the bladder wall being used to predict the bladder volume, it was important to determine the optimal site for placing the potentiometric module. A fresh pig bladder model was first utilized to establish the optimal site for implanting the potentiometer. To preserve the physiological properties of isolated fresh pig bladders in a normal condition, bladders were preserved in a 37 °C organ bath of calcium-free Tyrode’s solution bubbled with 95% O_2_ and 5% CO_2_ before all experimental trials. Typically, the maximal capacity of a human bladder is approximately 500 mL, and the volume that should trigger voiding in humans is 400 mL. Pig bladders were chosen as the experimental model, because the pig model has gained great popularity as the main species for developing implantable bladder sensing devices; and the size of the pig bladder capacity is approximately 500–550 mL, which is similar to that of humans.

**Figure 3:**
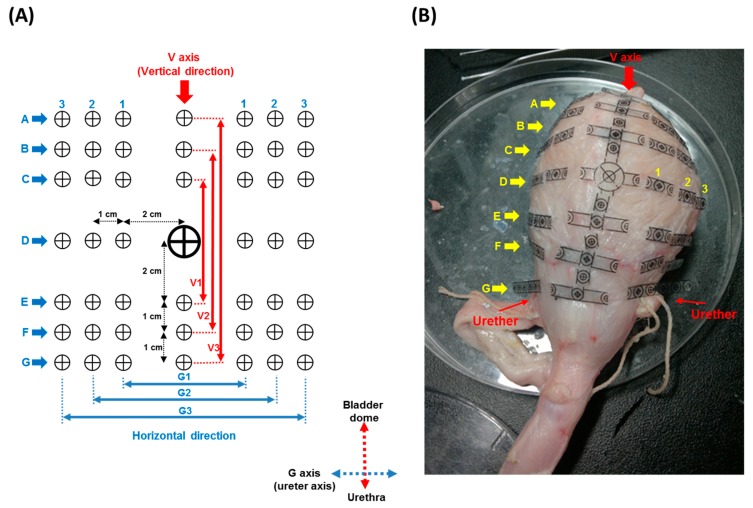
Geometric relationship between the bladder capacity and bladder wall length as quantified by a marker array label system. (**A**) The marker array consisted of one vertical axis (V axis) and seven horizontal axes (from A–G), in which each axis had three measured intervals; e.g., Axis G comprised the G1, G2 and G3 intervals; (**B**) each marker point was stuck onto the outer dorsal bladder wall with tissue adhesive. Axis G was defined as the line of two ureter points, and Axis V was defined as the midline of the dorsal bladder wall. The relationship between the bladder capacity and bladder wall length was established by repeatedly filling the pig bladder with saline from 0–500 mL in volume.

Thus, the geometric relationship between bladder capacity and bladder wall length was established with pig bladders (*n* = 6) via a marker array label system. The marker array was designed and made of transparent polyethylene terephthalate (PET) material. Each marker point was stuck onto the outer bladder wall with tissue adhesive (No. 1469, 3M Health Care, St. Paul, MN, USA). The marker labels were used to quantify changes in the horizontal and vertical directions of the pig bladder wall in response to various bladder capacities. The distance between any two markers was fixed to be 1 or 2 cm, as shown in Figure 3. The horizontal direction of the marker array system consisted of seven axes (Axes A–G), and each axis had three measured intervals. For example, Axis A comprised the A1, A2 and A3 intervals and Axis G the G1, G2 and G3 intervals (Figure 3). Note that the anatomic location of Axis G was defined as the line between the two ureter outlet points. The vertical direction only comprised one axis (Axis V), which was at the location of the midline of the dorsal side of the bladder, and three lengths (V1, V2 and V3). The relationship between the bladder capacity and bladder wall length was established by repeatedly filling the pig bladder with saline, through a polyethylene catheter (PE-160) inserted into the bladder through the urethra, and normal saline was infused into the bladder at 25 mL/min until the bladder capacity (500 mL) was reached. All measured lengths in each axis were recorded during the bladder filling trails. The length-volume relationship of all data was established with a polynomial fit performed by Microsoft Excel 2013^TM^ (Microsoft Corp., Redmond, WA, USA).

### 2.3. In Vitro Bladder Testing

Another six fresh pig bladders were used to assess whether the prototype potentiometric device could correctly detect the bladder capacity using this fresh pig bladder model. The potentiometric module was attached at the optimal location on the external bladder wall, the location of which was determined by results obtained from the geometric modeling pig bladder experiment. The performance of the prototype device was recorded at bladder volumes of 0–400 mL at 50-mL increments. The accuracy of the volume prediction was evaluated by calculating the ratio of the infusion volume to the volume detected by the prototype device. All procedures for the preservation of fresh pig bladders and monitoring of the bladder volume were performed similarly to the procedures described in the geometric modeling experiment.

## 3. Results

### 3.1. Basic Structure of the Prototype Potentiometric Platform

**Figure 4:**
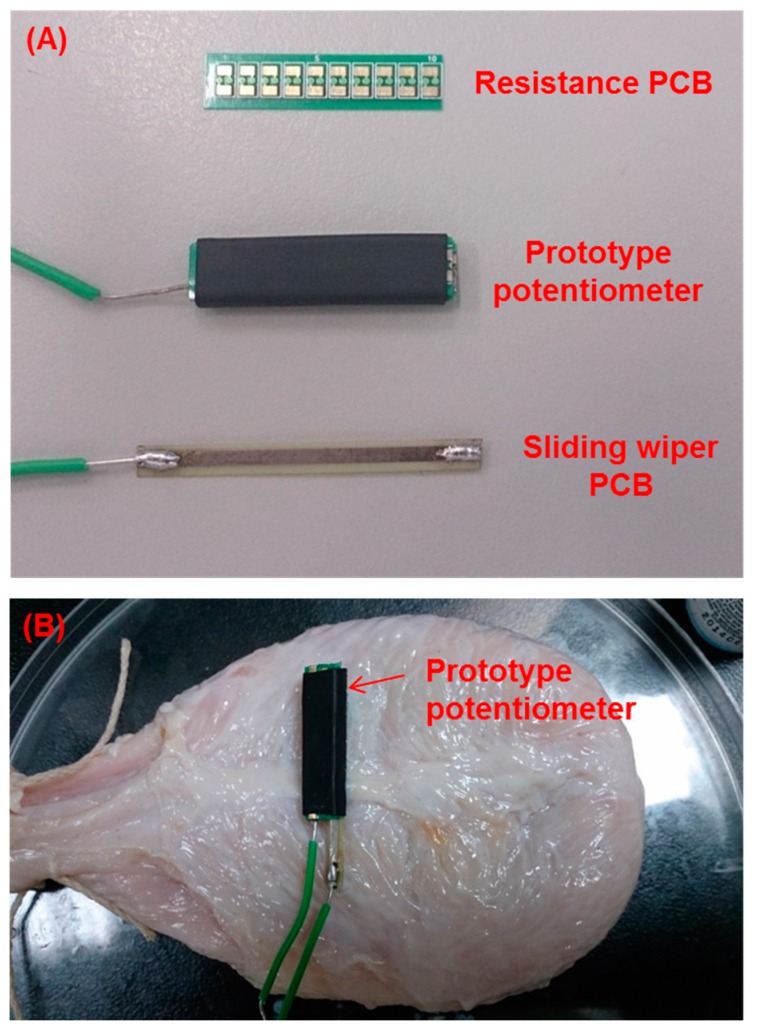
Configuration of the prototype potentiometric module. (**A**) The prototype potentiometric module was fabricated with two fiberglass printed circuit boards (PCBs), including a resistance and a sliding wiper PCB; (**B**) the packaged potentiometer module was fixed onto a fresh pig bladder for volume detection.

Figure 4 shows the configuration of the prototype potentiometric module, which was fabricated with two fiberglass PCBs. The size of the green PCB was designed for potentiometric resistance and was fabricated with nine 0805 SMD resistors on a single-layer PCB with dimensions of 2.4 cm × 0.56 cm. The yellow board functions as a sliding wiper of the potentiometer (with dimensions of 3.3 cm × 0.28 cm). Detailed specifications and components of the prototype potentiometric module are given in Table 1.

**Table 1:** Specifications for components of the potentiometric module.

Components	Length × Width (cm)	Thickness (mm)	Weight (g)
Resistance PCB	2.4 × 5.6	0.28	0.3
Sliding wiper PCB	3.3 × 2.8	0.24	0.4
Packaged potentiometer	2.4 × 6.6	3.3	0.9

PCB, printed circuit board.

The wireless transmission module and power unit were separately packaged and connected to this prototype potentiometric module. A Bluetooth wireless transmission module, an Arduino Nano 3.0 (ATMEGA328, Gravitech LLC, Minden, NV, USA), was used to send the digital data to an external receiver via wireless transmission. The received signal was further processed via the LabVIEW program (National Instruments, Austin, TX, USA), and bladder capacity information was displayed in real time on a laptop screen. The power supply to maintain the electronic circuit of the potentiometer and wireless transmission module was a 3-mA constant-current source from a commercial lithium battery. The total capacity of the battery was 2000 mAh, which was sufficient to power the prototype potentiometric platform to carry out the *in vitro* pig bladder trials. The power unit consisted of two current regulatory diodes connected in parallel. In cases of voltage fluctuations from 1–10 V or impedance fluctuations, it could still supply a stable electrical current.

### 3.2. Bladder Geometric Model

On the basis of our observations (*n* = 6 of six pig bladders), the dorsal side of the bladder wall was better than the ventral side for modeling the volume-length relationship, since the ventral side of the bladder wall did not exhibit significant changes in tissue length in any measured interval, even when the bladder capacity had reached a 400–500-mL condition. The low extensibility of the ventral bladder wall might have been due to the bladder morphology on the ventral side being thicker than that on the dorsal side, based on our experimental observations. Thus, the marker array labels were placed on the dorsal side of the bladder wall.

**Figure 5:**
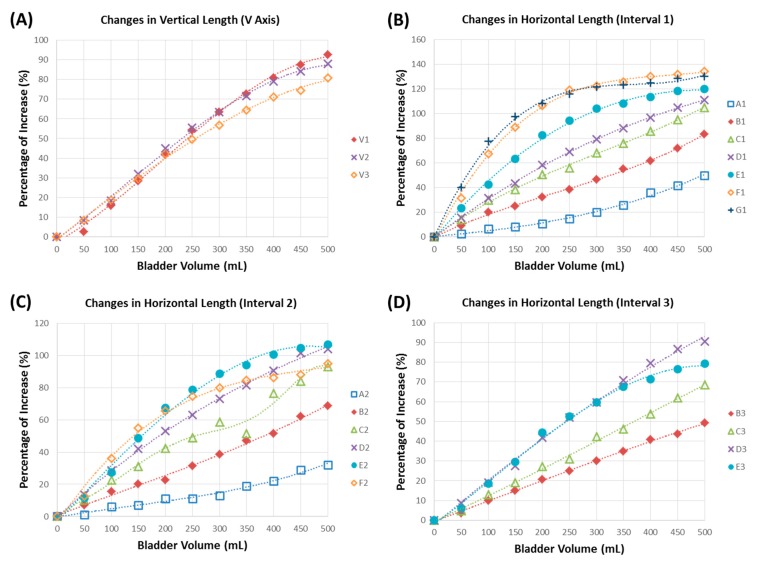
Results of the geometric relationship between the bladder capacity and normalized bladder wall length in the vertical and horizontal directions, including (**A**) the vertical length (axis V), (**B**) Interval 1, (**C**) Interval 2 and (**D**) Interval 3 for all horizontal axes. The percentage of increase represents the increased bladder wall length normalized relative to its original length. All data were further plotted using a polynomial (third-order) curve fitting with *R*^2^-values of at least 98.54%.

Results of the bladder wall lengths in the horizontal and vertical directions in response to bladder capacities of 0–500 mL are shown in Figure 5. Relationships for each measured length were well approximated with a third-order polynomial fit with *R*^2^-values of at least 98.54%. The measured lengths of V1, V2 and V3 in the vertical axis (V axis) all exhibited a maximal 80%–90% length increase when the bladder capacity was 500 mL, as shown in Figure 5A. However, the volume-length relationship of the V1, V2 and V3 intervals did not exhibit a good linear relationship, especially at bladder capacities of approximately 300–500 mL, and the measured intervals all exhibited a curved-shape pattern. Therefore, the V axis was not an optimal direction for implementing potentiometric measurements.

On the other hand, as for the results in the horizontal direction among the A1–G1 intervals, B1, C1 and D1 all exhibited good linear properties under various bladder capacities compared to the results of the A1, E1, F1 and G1 intervals (Figure 5B). The B1 interval exhibited good linear properties, and its maximal length increase was only 83%, which was lower than those of the C1 and D1 intervals with 105%–111% extensible properties. Thus, among the A1–G1 intervals, C1 and D1 were better than other sites for potentiometric measurements. For intervals A2–F2, the D2 interval had better linear and extensible properties than other corresponding intervals (Figure 5C). Note that we did not measure G2 (Figure 5C) or intervals A3, F3 or G3 (Figure 5D), because those markers were on the ventral side of the bladder wall. For results of the B3–E3 intervals, except for the E3 interval, all intervals exhibited linear-like properties, but interval D3 showed a better extensible property than the other intervals. To sum up, gathering all data from the geometric experiment, the D axis was the optimal axis for implementing the potentiometric module to detect the bladder capacity.

### 3.3. In Vitro Bladder Testing

The potentiometric module was placed on the middle point of Axis D in the dorsal bladder wall, which was based on the results of the bladder geometric experiments. Our data showed that errors for predicting the bladder capacity were all <3%, as evidenced by the ratio of the infusion volume to the detected volume, which ranged 97.1%–101.7% (Table 2).

**Table 2:** Accuracy of volume detection by the potentiometric module.

Infusion Volume (mL)	Volume Detection (mL)	Infused Volume/Volume Detected (%)
49 ± 6	50	98.00
101 ± 18	100	101.00
148 ± 20	150	98.90
203 ± 20	200	101.70
243 ± 24	250	97.10
299 ± 18	300	99.70
350 ± 31	350	99.90
395 ± 21	400	98.80

Values are the mean ± SD; *n* = 6 pig bladders.

## 4. Discussion

Although various sensing approaches for detecting bladder volume were investigated in previous studies [8,9,10,11,12,13,14,15,16,17,21,22], those sensors are still incapable of clinic usage, because of challenging problems. For example, the magnetic approach implants a permanent magnet and a magnetic field of a sensing element on the bladder and abdominal external walls, respectively. The merit of the magnetic approach is its simplicity of construction and low power consumption. However, accuracy errors are large because the Earth’s magnetism and peripheral magnetic fields all possibly affect its performance [8]. The impedance approach measures the impedance between opposing electrodes sutured diametrically to the bladder’s external wall. Many disadvantages hamper its clinical application; e.g., the sensitivity of impedance measurements degrades when the volume condition increases [9,21]. Moreover, the electrical current supplied to the electrodes may directly stimulate contractions of the detrusor muscle.

Bladder volume can be measured with ultrasound (US) instruments by detecting the nonlinear wave distortion from tissues and water. US devices have been widely used in hospitals due to the merits of this non-invasive measurement procedure. However, current portable US devices usually measure bladder volume in an intermittent way, which is still unsuitable for chronic or continual use by patients in the home or ambulatory environment. ENG measurements from bladder afferent nerves may be a new possible direction for the chronic monitoring of bladder volume, but a functional portable device has yet to be developed, since it requires complex signal processing and recognition [13,14,22].

In contrast to the above-mentioned techniques, the potentiometric bladder sensor is a new concept for monitoring bladder capacity. Our preliminary results demonstrated that the prototype potentiometer could correctly monitor the bladder capacity with an error of <3%. The accuracy of the prototype potentiometer should be tolerable and have a chance for clinical application, since the normal bladder volume for naturally triggering voiding is approximately 400 mL, and a volume at above 550 mL may lead to overdistension injury. However, it is expected that the use of the current prototype device in long-term implantation would encounter some difficulties.

The first difficulty is the size of the potentiometric sensor for surgical implantation. In our preliminary results, the size of the PCB-made sensor was only about 5 cm^3^, but the wireless transmission module and power unit were not integrated on the PCB sensor. Therefore, this prototype device may be too large for implantation inside of living animals. In the next stage, we need to integrate all of these elements into a single integrated-circuit sensor to develop a fully-functioning microelectromechanical system (MEMS) [23,24]. However, our potentiometric sensor was designed to be placed on the bladder outer wall, and thus, it is expected that the size restriction for implanting such a sensor will be more flexible than other bladder sensors that are totally implanted inside the bladder cavity [25,26]. We propose that the optimal shape of the implantable sensor should be a thin capsule of <10 cm^3^, which would allow for easy implantation of the device on the bladder outer wall with minimally invasive surgery from the abdominal wall.

Second, the power issue for chronic implantation must be also taken into consideration. For this preliminary study, the potentiometric and wireless transmission modules were powered by a lithium battery to provide 3 mA, which is not suitable for chronic implantation experiments. There are two feasible ways to improve power management of the sensing system for long-term implantation, including reducing power consumption and using an inductively-powered source [25,27]. Power consumption of the device can be greatly improved by optimizing the electronic circuits’ design with MEMS technology and developing an ultra-low-power-consuming device or by reducing the sampling rate of the telemetry module [25,26]. On the other hand, the inductive powering approach was reported in recent studies [26,27], which is a more feasible method than traditional batteries for long-term powering of neuroprosthetic implants.

Third, the mechanical limitations of the potentiometric scheme would be another challenge for implementation in chronic implantation experiments. This could result from two possible mechanical factors: (1) the movement of the sliding wiper board may be mechanically limited by surrounding visceral tissues; and (2) frictional resistance forces between the two PCB surfaces may be produced during sliding. For the first mechanical factor, encapsulating the potentiometric module in biocompatible glass may be a possible solution to this limitation. The two PCBs of the potentiometer could be sealed in a long capsule-like glass chamber to avoid mechanical influences on surrounding tissues. A small slit at the bottom of the glass chamber would allow the pin tip of the sliding wiper board to be sutured to the bladder wall. In addition, the glass chamber would have extra space to allow for displacement of the sliding wiper board as the bladder wall elongated. The small slit at the bottom of the glass chamber could be waterproofed to create a biocompatible package for the electronic circuitry [25,27,28] and to prevent protein adsorption onto the surface of the device, which would subsequently cause inflammation problems [29]. In addition, encapsulating the potentiometric module in a glass chamber could also prevent the surrounding tissue’s damage due to the movement of its sharp edges.

The prototype potentiometric module was successfully implemented in the present experiment, which demonstrated that the sliding wiper board was not significantly limited by the existence of frictional force between the two PCB surfaces. However, the issue of frictional forces should be taken into consideration during the manufacturing of a sophisticated implantable potentiometer in the future. Friction force could be reduced by coating with a material with a low coefficient of friction onto the contact areas between the two PCBs, such as steel materials. In addition, the application of electrically-conductive lubricants to the contact areas between the two PCBs would be another possible way to lessen the coefficient of friction.

Finally, calibration issues should also be included in future implant experiments. Although the error for predicting the bladder capacity was <3% in the present *in vitro* experiment model, this experiment did not consider the influence of biological hysteresis. Biological hysteresis is a phenomenon of the tension of the bladder muscle held at great length after stretching, gradually decreasing and eventually falling at or below the level of that before being stretched. Tension length or pressure volume hysteresis was demonstrated in isolated and intact animal and human bladders [30,31]. The response to bladder hysteresis would be dependent on a number of factors, such as the duration and rate of stretching and sympathetic/parasympathetic nerve interactions. However, our potentiometric scheme for monitoring bladder volume was mainly determined by the length-volume relationship, which should be less affected by a hysteresis effect compared to using bladder pressure for predicting bladder volumes. Nevertheless, we cannot exclude the possibility of biological hysteresis influencing the accuracy of volume detection in chronic animal experiments, and the possibility warrants further exploration.

In the bladder geometric study, the dorsal side of the bladder was determined to be the optimal location for positioning the potentiometric module. This result would be consistent with LUT conditions in humans. This is because when the human bladder fills with urine, it generally assumes an ovoid or even spherical configuration and ascends into the abdominal cavity along the anterior abdominal wall [32]. Therefore, implanting the potentiometric sensors on the bladder dorsal side could avoid mechanical compression due to bladder fullness. Our results demonstrated that most of the measured axes exhibited a linear relationship between the bladder tissue length and bladder capacity (Figure 5). However, the maximal increase in tissue length at 500 mL of bladder capacity exhibited a large variation among all measured intervals, in which the range of the increased length was 32%–134%. Axis D in the horizontal direction (4 cm below the bladder dome) was the optimal site for implementing the potentiometric scheme, since all measured intervals had at least >80% of the maximal length increase and nearly linear properties.

Although we used a polynomial curve fitting technique to perform bladder volume length calibration, results of the capacity-length relationship were not all a perfectly straight line, and a decreasing trend in tissue length was even detected during continuous bladder infusion (e.g., Line C2 in Figure 5C). This phenomenon might have resulted from some areas of the bladder surface not being completely smooth, and thus, certain segments of the capacity-length curve exhibited non-linear properties. In addition, the bladder shape may considerably differ in normal and pathological conditions, which may increase the difficulty of calibrating the potentiometric scheme. Again, in future experiments, calibration issues should be taken into consideration. The non-linear problem could be corrected through a more suitable calibration process, such as with advanced biomedical signal processing or statistical regression approaches. To the best of our knowledge, few studies have explored the geometric dimensions of pig bladders in response to volume changes. Our study is the first to report on quantification of the geometric dimensions of pig bladders. These results can be utilized as useful information for real-time monitoring of the bladder capacity in future *in vivo* experiments.

## 5. Conclusions

In the present study, we successfully designed and proved a new simple concept of bladder capacity monitoring using a flexible potentiometric scheme in an *in vitro* pig bladder model. Our results showed that the prototype potentiometric device performed well at a location on Axis D of the dorsal bladder wall, where the error of the accuracy of volume detection was <±3%. However, it is unclear whether the results of the potentiometric scheme for bladder capacity monitoring can be translated into chronic implant experiments, and thus, further research is needed to confirm this.
